# Among the Colleges

**Published:** 1897-07

**Authors:** 


### AMONG THE COLLEGES.

The Royal Veterinary College Council have declined recognition of the Melbourne Veterinary College.

English veterinarians are not exempt from jury duty. A special Parliamentary Act will be required, which is one of the future legislative moves planned.

Three English veterinarians have obtained the fellowship degree by examination. Of nearly 200 applicants but 119 were successful in obtaining the diplomas of membership in the Royal College of Veterinary Surgeons.

The University of Buffalo has just established a Chair of Comparative Pathology. Gradually, but surely, the world is growing to appreciate the true importance of comparative science.

Just on the eve of going to press we learn of another veterinary college entering the field of work, to be located at Kansas City, Missouri, to be known as the Western Veterinary College, and its moving spirit, Dr. Junius H. Wattles, M.D., D.V.S. It proposes to have as complete a course scientifically as any college in America and to comply with all the requirements of the United States Veterinary Medical Association. The degree of (D.V.S.) Doctor of Veterinary Science is to be conferred. Six months' sessions to be maintained. In the absence of other information we are wholly at a loss to comprehend the need of two schools in that city or in that section. The Kansas City Veterinary College, organized by Dr. Wattles, was relinquished, we thought, because of failing health and a sojourn southward being necessary. Subsequently the selling of the Doctor's practice was recorded, and later on the announcement of his entrance upon human medicine as a study for future work. Surely there cannot be a demand so soon for an additional school in that territory, in view of the light classes at the Western veterinary colleges last year, and we hope ere another issue to learn more of the situation. In the East we are hopeful for the amalgamation of some of our schools in order that stronger teaching institutions may be developed, and Western movements have been noted looking to the same wise end.

